# YOLOv12-VSD: A Transfer-Learning-Assisted Real-Time Detection Algorithm for Vehicle Surface Defects

**DOI:** 10.3390/s26092905

**Published:** 2026-05-06

**Authors:** Haopu Liu, Dequn Zhao, Yu Li

**Affiliations:** 1 Beijing Dublin International College, Beijing University of Technology, Beijing 100124, China; 2 School of Information Science and Technology, Beijing University of Technology, Beijing 100124, China

**Keywords:** object detection, vehicle surface defect, deep learning, YOLOv12, IoU-aware classification loss, structural reparameterization, transfer learning

## Abstract

Vehicle surface defect detection faces three core challenges: classification–localization inconsistency for boundary-sensitive defects, insufficient multi-scale feature response across defect sizes, and cross-scenario generalization degradation caused by domain shift among production lines. This paper proposes YOLOv12-VSD, an improved detection algorithm addressing these issues through coordinated modifications at three levels. An IoU-aware classification loss aligns classification confidence with localization quality. A reparameterized convolution module at the P4 feature level (P4-RepC3) enriches intermediate-layer directional feature diversity without increasing inference cost. A multi-scale spatial pyramid pooling–fast structure at the P5 feature level (P5-SPPF) expands the effective receptive field for large-area defects. A three-stage transfer learning framework comprising source-domain pretraining, target-domain adaptation, and low-learning-rate refinement is further designed to reduce domain shift with limited annotations. Experiments show that YOLOv12-VSD achieves a mean Average Precision at IoU threshold 0.50 (mAP@50) of 0.715, the highest among six comparison models, with only 6.1M parameters and 17.1 giga floating-point operations per second (GFLOPs). After three-stage transfer, mAP@50 improves from 0.531 to 0.652, with training duration reduced by 64%.

## 1. Introduction

Vehicle surface defect detection is a critical step in automotive end-of-line quality inspection. Undetected defects such as cracks, paint peeling, dents, and corrosion directly affect product appearance and durability, incurring rework costs and brand reputation losses [1]. Traditional manual inspection suffers from low efficiency and strong subjectivity, while threshold-based image processing methods lack robustness under specular reflection and complex paint texture conditions. Deep-learning-based detection models have substantially improved recognition accuracy and real-time performance for this task [2,3]. Nevertheless, three core challenges persist: (1) boundary-sensitive defects with high aspect ratios (e.g., elongated cracks) cause regression deviation and classification–localization inconsistency; (2) the scale span from minute pitting to large-area peeling demands both fine-grained and global feature perception; and (3) domain shift among vehicle models, equipment, and inspection stations constrains cross-scenario generalization.

Accurate localization and classification of boundary-sensitive defects remain difficult. Two-stage detectors such as Faster R-CNN achieve high accuracy through region proposal and refinement [4], and recent variants have introduced deformable convolutions [5] and multi-scale attention [6] to improve boundary regression for irregular defect shapes. However, their cascaded architectures incur high per-frame latency, limiting real-time applicability on production lines. One-stage YOLO detectors offer a favorable speed–accuracy trade-off for industrial deployment [7]. Wang et al. [8] improved YOLOv3 with focal loss to mitigate class imbalance in paint defects, yet the backbone’s limited expressiveness led to high false positive rates. Jiang et al. [9] enhanced boundary regression in YOLOv7 through residual multi-branch modules, at the cost of increased training overhead. Despite these efforts, a common limitation persists: classification confidence is decoupled from localization quality, causing high-confidence predictions with poor boundary fit—a critical issue for elongated cracks where even slight misalignment substantially degrades IoU [10].

The scale diversity of vehicle surface defects adds another layer of difficulty, with damage ranging from minute pitting to large-area paint peeling. Li et al. [11] introduced lightweight attention and adaptive fusion into YOLO to improve efficiency for multi-scale defects, but the compressed high-level features degraded detection of large-area damage. Liu et al. [12] combined YOLOv5 with deflectometry to enhance paint defect contrast, though hardware dependence limits deployment flexibility. Zhang et al. [13] applied deformable convolution and dynamic label assignment in YOLOv8, achieving strong results on steel defects but without validated cross-domain generalization to automotive surfaces. Wei et al. [14] used the Swin Transformer for long-range contextual modeling of large defects, yet the quadratic attention complexity constrains real-time operation [15]. YOLOv12 [16], a recent high-performing YOLO iteration with area attention, improves general detection but lacks explicit multi-scale pooling at the P5 layer and sufficient directional diversity at the P4 layer for vehicle-specific defect patterns.

Domain shift across production lines, vehicle models, and acquisition equipment degrades detection performance in cross-scenario deployment [17,18]. Transfer learning mitigates this by reusing source-domain representations under limited target-domain annotation conditions [19]. Kim et al. [20] applied a pretrain–fine-tune paradigm with YOLOv5, shortening training cycles but achieving limited generalization with large domain gaps due to a single fixed-rate fine-tuning step. Gao et al. [21] designed a two-stage transfer pipeline for stamped part defects using YOLOv4 with manual freezing-depth tuning, which lacks adaptability across different vehicle models. Park et al. [22] combined domain adversarial training with YOLO to enforce domain-invariant features, though optimization instability with large domain gaps limits practical effectiveness. Fu et al. [23] integrated multi-scale transfer with YOLOv8 for glass defect detection, improving fine-defect recall at the cost of increased complexity. A common shortcoming of existing transfer approaches is the reliance on a simple two-step pipeline (pretrain then fine-tune) without hierarchical adaptation, leading to either catastrophic forgetting or insufficient domain alignment [24,25].

The overarching goal of this work is to achieve fast, high-accuracy defect detection across diverse inspection scenarios—a single problem that decomposes into two sub-problems: (1) how to construct a detection model that attains high accuracy on boundary-sensitive and multi-scale vehicle defects and (2) how to adapt such a model to new production lines with minimal annotation and training time. These two sub-problems motivate, respectively, the algorithm design and the transfer learning framework proposed below. The YOLO architecture is chosen as the backbone because its single-pass inference provides the real-time throughput required by production-line deployment, where Transformer-based alternatives incur prohibitive latency. The main contributions are:

1. To address sub-problem (1)—high-accuracy detection of boundary-sensitive and multi-scale vehicle defects—the YOLOv12-VSD algorithm is proposed with three targeted modifications, each driven by a specific domain-level deficiency rather than by heuristic combination: (a) classification–localization decoupling in boundary-sensitive defects (e.g., elongated cracks) motivates an IoU-aware classification loss that injects localization quality into sample assignment and classification targets, suppressing high-confidence but poorly localized predictions; (b) insufficient directional feature diversity at the P4 layer for mid-scale defects (e.g., cutouts, localized paint damage) motivates P4-RepC3, a reparameterized convolution module that enriches mid-layer filter diversity during training while folding into a single convolution at inference for zero additional cost; (c) absence of explicit multi-scale pooling at the P5 layer for large-area defects (e.g., paint peeling, pitting) motivates P5-SPPF, a lightweight spatial pyramid pooling structure that expands the effective receptive field through cascaded spatial pooling. The three modifications are not independently arbitrary choices but coordinated responses to three distinct failure modes observed in the baseline.
2. To address sub-problem (2)—rapid cross-scenario adaptation under limited annotation conditions—a three-stage transfer learning pipeline is designed comprising source-domain pretraining (Stage I), target-domain high-rate adaptation (Stage II), and target-domain low-rate refinement (Stage III). Stage II is initialized from Stage I weights and rapidly aligns feature distributions to the target domain; Stage III is initialized from Stage II weights and conservatively refines discriminative boundaries with a reduced learning rate. This progressive strategy balances rapid domain alignment with preservation of general features, reducing performance fluctuation and training time in cross-scenario deployment.

## 2. YOLOv12-VSD Algorithm

YOLOv12 achieves competitive performance on general object detection benchmarks. However, when applied to vehicle surface defect detection, three domain-specific issues emerge. Elongated cracks and curved scratches with high aspect ratios are sensitive to boundary fitting accuracy, yet the classification branch of YOLOv12 does not encode localization quality, causing inconsistency between classification confidence and actual IoU. Mid-scale defects such as cutouts and localized paint damage require rich directional feature responses, but the A2C2f topology at the P4 layer offers limited convolutional diversity. Large-area peeling and pitting against complex automotive paint backgrounds demand broad contextual modeling, which the P5 layer cannot provide without explicit multi-scale pooling.

**Figure 1:**
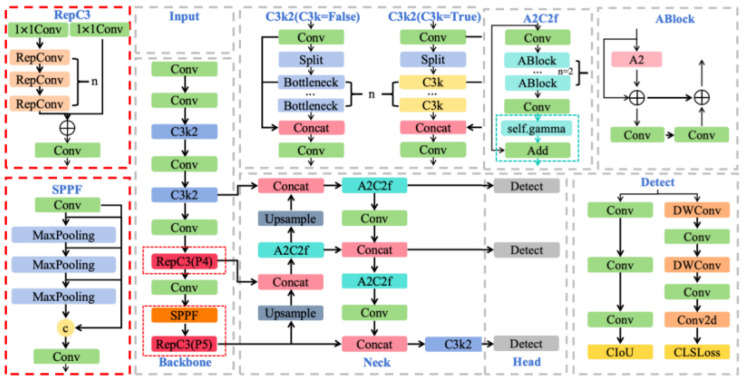
Architecture of YOLOv12-VSD. The red dashed box highlights the two novel modules (P4-RepC3 and P5-SPPF) introduced relative to the original YOLOv12 baseline.

To address these three issues, the improved model YOLOv12-VSD is proposed with targeted modifications at three levels. An IoU-aware classification loss injects localization quality into sample assignment and classification targets, suppressing high-confidence but poorly localized predictions. A reparameterized convolution module deployed at the P4 feature level (P4-RepC3) enriches mid-layer filter diversity through multi-branch training while folding into a single convolution at inference for zero additional cost. A multi-scale spatial pyramid pooling–fast structure at the P5 feature level (P5-SPPF) expands the effective receptive field through cascaded spatial pooling, enhancing discriminability for large-area defects. The overall framework is shown in Figure 1.

### 2.1. YOLOv12 Baseline

**Figure 2:**
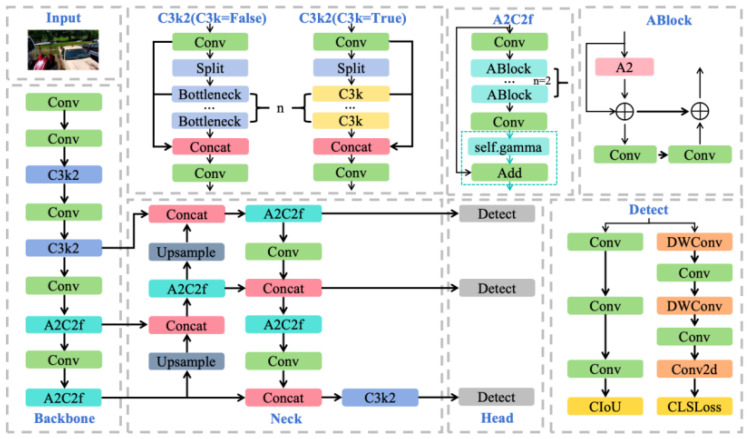
Architecture of YOLOv12.

YOLOv12 [16] is a recent high-performing iteration of the YOLO single-stage detection family [3], building upon YOLOv9 [26] with area attention and reparameterized convolution. The nano-scale variant (YOLOv12-N) was selected as the architectural baseline because it provides a compact and efficient single-stage foundation for real-time inspection while satisfying the memory and latency constraints typical of automotive production-line hardware. It also exposes several task-relevant bottlenecks in classification-localization consistency and multi-scale feature representation, making it a suitable starting point for the proposed targeted improvements. In addition, adopting YOLOv12 as the unified base model facilitates controlled ablation, fair comparison, and integration with the three-stage transfer learning framework. As shown in Figure 2, it retains the backbone–neck–head architecture. The backbone extracts multi-level features through hierarchical convolutions with cross-stage partial connections. The neck performs multi-scale fusion via feature pyramid and path aggregation [27]. The detection head adopts an anchor-free decoupled design, predicting bounding boxes, categories, and confidence scores at multiple scales.

Despite its strong general-purpose performance, YOLOv12 exhibits three limitations when applied to vehicle surface defects. (1) The CIoU-based regression loss is decoupled from the classification loss, resulting in weak correlation between classification confidence and localization quality. (2) The A2C2f stacking at the P4 layer provides limited directional diversity, constraining mid-scale feature expressiveness. (3) The P5 layer relies on standard convolutions without explicit multi-scale pooling, limiting contextual modeling for large-area defects.

### 2.2. IoU-Aware Classification Loss

Vehicle surface defects such as elongated cracks (aspect ratios up to 20:1), curved scratches, and irregular paint peeling are highly sensitive to boundary fitting accuracy. A slight misalignment between the predicted box and the actual defect contour can cause the detector to assign a high classification score to a poorly localized prediction. This arises because YOLOv12 decouples classification from regression: the classification branch learns only category discrimination without encoding localization quality. As a result, high-confidence predictions may correspond to low IoU, degrading non-maximum suppression and increasing false positives.

To resolve this inconsistency, the IoU-aware mechanism [28] is introduced. Its core idea is to use the IoU between each prediction box and its matched ground-truth box as a quality prior, injecting it into both sample assignment and classification loss. This ensures that classification scores maintain a monotonically positive correlation with localization precision.

This modification does not redesign the regression head. Instead, it operates at the supervision level by injecting IoU-derived quality information into sample assignment and classification target/loss construction.

Given a prediction box *B* and a ground-truth box *G*, their IoU is defined as

(1) $$\mathrm{IoU}\left(B,G\right)=\frac{\left|B\cap G\right|}{\left|B\cup G\right|}$$

CIoU (Complete IoU) [29] extends IoU with center-point distance penalty and aspect ratio consistency constraints and was adopted as the regression metric. The boundary regression loss is

(2) $$L_{\mathrm{IoU}}=\frac{1}{N}\sum_{i=1}^{N}\left(1-\mathrm{CIoU}(B_{i},G_{i})\right)$$

where *N* is the total number of positive samples.

In the positive–negative sample assignment stage, the model follows the Task-Aligned Assignment (TAA) [30] strategy. The task alignment metric can be expressed as

(3) $$A_{ij}=p_{ij}^{\gamma }\cdot \mathrm{IoU}{\left(B_{i},G_{j}\right)}^{\delta }$$

where $p_{ij}$ is the predicted confidence that anchor point *i* belongs to the *j*-th defect class. γ and δ are weight coefficients.

Let $G_{i}$ denote the ground-truth box to which anchor point *i* is ultimately assigned and its one-hot category label be

(4) $$y_{i}\in {\left\{0,1\right\}}^{C}$$

After completing positive sample assignment, IoU quality weights are introduced on top of the one-hot category labels, constructing a quality-aware target vector:

(5) $$q_{i}^{c}=\mathrm{IoU}\left(B_{i},G_{i}\right)\cdot y_{i}^{c}$$

The classification branch is optimized using binary cross-entropy loss with $q_{i}^{c}$ as soft labels:

(6) $$L_{\mathrm{cls}}=\frac{1}{S}\sum_{i=1}^{N}\sum_{c=1}^{C}\left[-q_{i}^{c}\log\sigma \left(p_{i}^{c}\right)-\left(1-q_{i}^{c}\right)\log\left(1-\sigma \left(p_{i}^{c}\right)\right)\right]$$

where *N* is the number of positive sample anchor points, *C* is the number of defect categories, and *S* is a normalization factor.

In addition to regression and classification losses, Distribution Focal Loss (DFL) [28] is employed to refine bounding box coordinates by learning a discretized probability distribution over candidate positions:

(7) $$L_{\mathrm{DFL}}=-\left(\left(y_{i+1}-y\right)\log\left(p_{i}\right)+\left(y-y_{i}\right)\log\left(p_{i+1}\right)\right)$$

where *y* is the continuous regression target, $y_{i}$ and $y_{i+1}$ are the two nearest discrete positions satisfying $y_{i}\leq y\leq y_{i+1}$, and $p_{i}$, $p_{i+1}$ are their predicted probabilities.

The complete loss function is

(8) $$L=\lambda_{\mathrm{box}}L_{\mathrm{IoU}}+\lambda_{\mathrm{cls}}L_{\mathrm{cls}}\left(p,q\right)+\lambda_{\mathrm{dfl}}L_{\mathrm{DFL}}$$

Through this design, classification scores are tightly bound to localization quality. Defects with accurate boundary fitting receive higher classification confidence, while poorly localized candidates are naturally suppressed. Importantly, the mechanism operates entirely at the loss level by reconstructing supervision labels and sample assignment criteria. It introduces no additional network parameters or inference-time computation.

### 2.3. P4-RepC3

Mid-scale vehicle surface defects, such as cutouts, localized paint damage, and short cracks, exhibit diverse geometric morphologies including irregular edges, varying orientations, and fine-grained texture gradients against specular automotive paint. Capturing these patterns requires rich directional and scale diversity in intermediate-layer convolution filters. However, the P4 layer of YOLOv12 employs an A2C2f module driven by area attention, which re-weights existing features but does not introduce explicit multi-directional convolutional decomposition. To address this limitation, the P4-RepC3 [31] reparameterized convolution module is introduced at the P4 layer.

Given the P4 input feature $X\in R^{C\times H\times W}$, the P4-RepC3 module first constructs complementary intermediate representations through two 1×1 convolution branches:

(9) $$U_{0}={\mathrm{Conv}}_{1\times 1}^{\left(1\right)}\left(X\right),\;V_{0}={\mathrm{Conv}}_{1\times 1}^{\left(2\right)}\left(X\right),\;U_{0},V_{0}\in R^{C^{\prime }\times H\times W}$$

The main branch $U_{0}$ passes sequentially through *n* RepConv units:

(10) $$U_{k}=\phi^{\left(k\right)}\left(U_{k-1}\right),\;k=1,\ldots ,n$$

During training, each RepConv unit consists of a 3 × 3 convolution branch and a 1 × 1 convolution branch in parallel:

(11) $$\phi^{\left(k\right)}\left(U\right)=\sigma \;\left(W_{3\times 3}^{\left(k\right)}*U+b_{3\times 3}^{\left(k\right)}+W_{1\times 1}^{\left(k\right)}*U+b_{1\times 1}^{\left(k\right)}\right)$$

After completing multi-layer RepConv stacking, P4-RepC3 performs element-wise residual fusion:

(12) $$Y=U_{n}+V_{0}$$

During inference, the dual-branch convolutions are equivalently folded into a single 3 × 3 convolution kernel:

(13) $$\phi_{\mathrm{infer}}^{\left(k\right)}\left(U\right)=\sigma \;\left(W^{\left(k\right)}*U+b^{\left(k\right)}\right)$$

where the equivalent parameters are

(14) $$W^{\left(k\right)}=W_{3\times 3}^{\left(k\right)}+\mathrm{pad}\left(W_{1\times 1}^{\left(k\right)}\right),\;b^{\left(k\right)}=b_{3\times 3}^{\left(k\right)}+b_{1\times 1}^{\left(k\right)}$$

**Figure 3:**
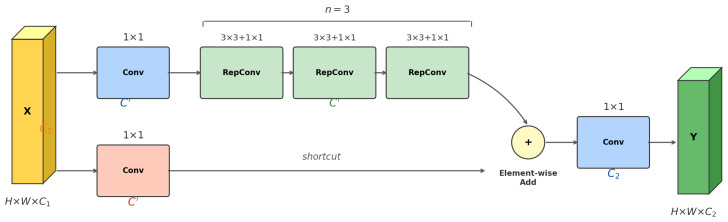
Schematic diagram of P4-RepC3 structure.

The schematic diagram of P4-RepC3 is shown in Figure 3.

### 2.4. P5-SPPF

Large-area defects on vehicle surfaces, including paint peeling spanning tens of pixels, extensive pitting clusters, and spidery glass fractures, require the detector to aggregate broad spatial context while preserving edge detail. These defects often appear against complex automotive paint backgrounds with specular highlights and color gradients, making them prone to background confusion when the receptive field is insufficient. The P5 layer of YOLOv12 relies solely on standard convolution stacking, which lacks explicit multi-scale contextual modeling. To address this, a lightweight P5-SPPF (SPPF [32] + RepC3 [31]) joint structure is introduced at the P5 layer.

For the input feature $X\in R^{C\times H\times W}$, SPPF first compresses the channel dimension:

(15) $$y_{0}={\mathrm{Conv}}_{1\times 1}^{\left(1\right)}\left(X\right),\;y_{0}\in R^{C^{\prime }\times H\times W},\;C^{\prime }=C/2$$

Three sequential pooling operations construct multi-scale contextual representations:

(16) $$y_{1}={\mathrm{MaxPool}}_{k}\left(y_{0}\right),\;y_{2}={\mathrm{MaxPool}}_{k}\left(y_{1}\right),\;y_{3}={\mathrm{MaxPool}}_{k}\left(y_{2}\right)$$

The features at four scales are concatenated along the channel dimension:

(17) $$Z=\mathrm{Concat}\left(y_{0},y_{1},y_{2},y_{3}\right)\in R^{4C^{\prime }\times H\times W}$$

A 1 × 1 convolution maps back to the original channel count:

(18) $$X_{\mathrm{SPPF}}={\mathrm{Conv}}_{1\times 1}^{\left(2\right)}\left(Z\right)\in R^{C\times H\times W}$$

Subsequently, $X_{\mathrm{SPPF}}$ is fed into the RepC3 block within the P5-SPPF module:

(19) $$Y_{P5}=f_{\mathrm{RepC}3}\left(X_{\mathrm{SPPF}}\right)$$

**Figure 4:**
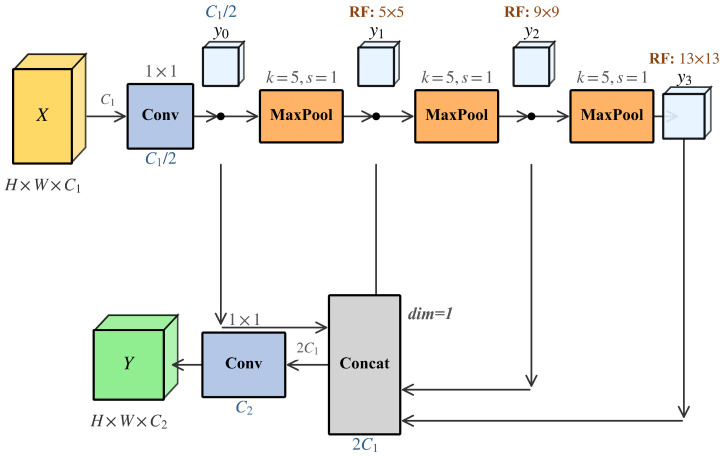
Schematic diagram of P5-SPPF structure.

The overall information flow is illustrated in Figure 4.

## 3. Three-Stage Transfer Learning Framework

In real-world vehicle quality inspection, imaging conditions vary considerably across production lines, vehicle models, and acquisition equipment. Differences in lighting, paint color, camera angle, and inspection station layout introduce significant domain shift between the data used for model training and the actual deployment environment [19]. Moreover, collecting and annotating large-scale defect samples for every new production scenario is costly and time-consuming. A staged fine-tuning strategy can mitigate these issues by first learning general defect representations on a data-rich source domain and then progressively adapting to the target domain under limited annotation conditions [24]. Accordingly, a three-stage transfer learning framework is designed around YOLOv12-VSD, comprising source-domain pretraining, target-domain adaptation, and low-learning-rate refinement.

### 3.1. Source and Target Domain Datasets

This study uses the NCAT12-DET vehicle surface defect dataset [33] as the source domain. The original image collection comprises over 20,000 images; a curated subset of 7200 high-resolution images was selected to ensure balanced coverage across all eight defect categories, using stratified sampling within each train/valid/test split to preserve the original class distribution. The subset covers eight typical vehicle surface states and defect types spanning three groups: glass surface defects (Clear Glass, Hairline Glass, and Spidery Glass), coating damage (Crack, Defaced Paint, and Paint-Peel), and structural anomalies (Cutouts and Inclusion).

**Figure 5:**
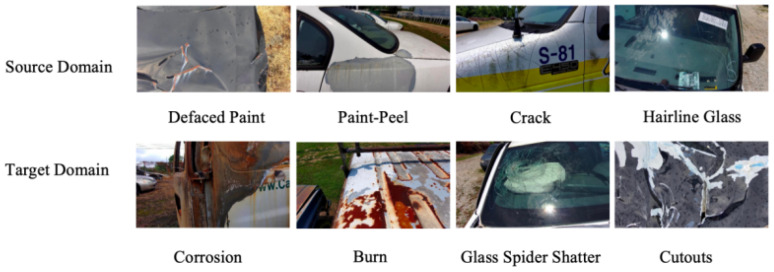
Partial image comparison between source-domain and target-domain data.

To further verify the model’s adaptability and effectiveness, a task-aligned target-domain evaluation set was derived from the publicly available CarDD dataset [34]. Starting from an initial target size of 2000 images, we first defined an eligible category pool based on semantic correspondence with the vehicle surface defect inspection task and then randomly sampled images within the official train/valid/test splits in proportion to the original split sizes. After annotation verification, 1998 valid images were retained. Category remapping was used only to align semantically comparable defect concepts across domains for transfer evaluation. The resulting target subset should therefore be interpreted as a task-aligned CarDD-derived evaluation set rather than a reproduction of the full category space and exact marginal distribution of the original CarDD benchmark. No image was reassigned across official splits, and no image-level overlap was introduced between the source- and target-domain subsets. A partial comparison of source-domain and target-domain images is shown in Figure 5.

### 3.2. Framework Design

**Figure 6:**
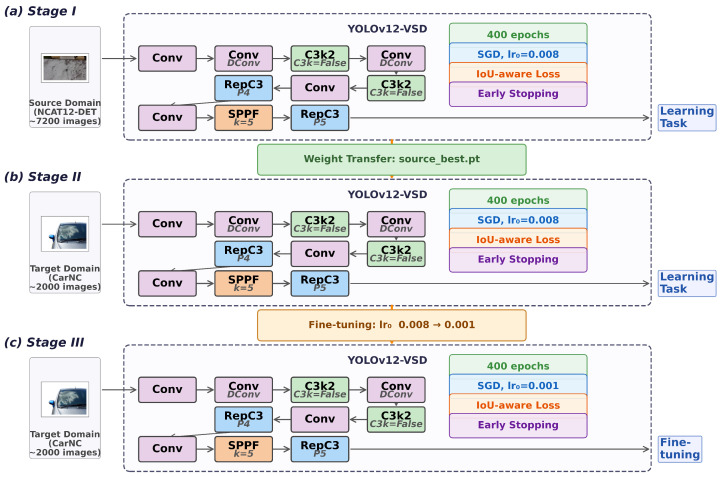
Schematic diagram of the three-stage transfer learning framework.

A naive two-step approach (pretrain then fine-tune) often leads to either catastrophic forgetting of source-domain features or insufficient adaptation to the target distribution. To balance feature retention and domain alignment, a three-stage framework was constructed. The schematic diagram is shown in Figure 6.

**Stage I: Source-domain pretraining** (Figure 6a). The model is trained on the large-scale source-domain dataset with a standard learning rate (0.008). The objective is to learn general defect representations, including local texture patterns (scratches and pitting) and geometric morphologies (cracks and peeling contours), that are shared across vehicle inspection scenarios. The resulting optimal weights serve as the initialization for the subsequent stage.

**Stage II: Target-domain high-rate adaptation** (Figure 6b). Starting from Stage I weights, the model is trained on the target-domain dataset with the same learning rate (0.008). The high learning rate enables rapid alignment of feature distributions to the new domain. Because all parameters are updated, the network can reorganize both low-level texture filters and high-level semantic representations to accommodate target-specific characteristics such as different paint colors, lighting conditions, and defect distributions.

**Stage III: Target-domain low-rate refinement** (Figure 6c). Initialized from Stage II weights, the model is further fine-tuned on the same target-domain data with a reduced learning rate (0.001). The low learning rate preserves the domain-aligned features acquired in Stage II while making conservative adjustments to the high-level discriminative boundaries. This progressive refinement is particularly beneficial for rare and hard-to-detect categories (e.g., hairline glass cracks), where aggressive parameter updates in Stage II may introduce instability. The best checkpoint within Stage III was selected as the final model.

### 3.3. Summary of Modifications Relative to Baseline YOLOv12

Table 1 provides a structured overview of all proposed modifications relative to the baseline YOLOv12, including the insertion position, purpose, and computational cost of each component.

## 4. Experiments and Analysis

### 4.1. Experimental Setup

The experiments were conducted on a high-performance cloud-based GPU server with an Intel Xeon Gold 6430 processor, an NVIDIA RTX 4090 GPU (24 GB), and 120 GB system memory. Python 3.12, PyTorch 2.5.1 with CUDA 12.4, and Ultralytics YOLO framework were employed. The specific configuration is listed in Table 2.

Training was performed with a batch size of 16 for 400 epochs, with input images resized to 640 × 640. The optimizer was SGD with an initial learning rate of 0.008, momentum of 0.937, and weight decay of 0.0005. Early stopping used a patience of 100 epochs, AMP was enabled, and the random seed was set to 0. Key augmentation and loss settings are summarized in Table 3, while unspecified options followed the default Ultralytics configuration.

### 4.2. Evaluation Metrics

Model performance is evaluated from two aspects: detection accuracy and computational efficiency. For accuracy, precision (P) and recall (R) characterize prediction correctness and defect coverage, respectively, with mAP@50 and mAP@50:95 serving as comprehensive metrics. For efficiency, GFLOPs measure inference-phase computational cost, FPS characterizes real-time inference throughput, and Training time records overall training overhead.

Precision (*P*) measures the proportion of true positives among all positive predictions:

(20) $$P=\frac{\mathrm{TP}}{\mathrm{TP}+\mathrm{FP}}$$

where TP is true positives and FP is false positives. Recall (*R*) measures the proportion of actual defects successfully detected:

(21) $$R=\frac{\mathrm{TP}}{\mathrm{TP}+\mathrm{FN}}$$

where FN is false negatives. mAP@50 averages the per-category average precision at IoU threshold 0.5:

(22) $$\mathrm{mAP}@50=\frac{1}{C}\sum_{c=1}^{C}{\mathrm{AP}}_{c}{|}_{\mathrm{IoU}=0.5}$$

where *C* is the number of categories and ${\mathrm{AP}}_{c}$ is the area under the precision–recall curve for class *c*. mAP@50:95 extends this by averaging over IoU thresholds from 0.50 to 0.95:

(23) $$\mathrm{mAP}@50:95=\frac{1}{C\cdot T}\sum_{c=1}^{C}\sum_{t=1}^{T}{\mathrm{AP}}_{c}{|}_{\mathrm{IoU}=\tau_{t}}$$

where $\tau_{t}\in \left\{0.50,0.55,\ldots ,0.95\right\}$ and *T* is the number of thresholds. GFLOPs normalizes the raw floating-point operation count to billions:

(24) $$\mathrm{GFLOPs}=\frac{\mathrm{FLOPs}}{10^{9}}$$

Training time records the wall-clock duration from initialization to training completion:

(25) $$T_{\mathrm{train}}=t_{\mathrm{end}}-t_{\mathrm{start}}$$

where $t_{\mathrm{start}}$ and $t_{\mathrm{end}}$ are the start and end timestamps, respectively.

Frames Per Second (FPS) quantifies inference throughput as the number of images processed per second under single-image batch conditions:

(26) $$\mathrm{FPS}=\frac{M}{T_{\inf}}$$

where *M* is the number of test images and $T_{\inf}$ is the total wall-clock inference time excluding data loading and result post-processing.

### 4.3. Training Process Analysis

**Figure 7:**
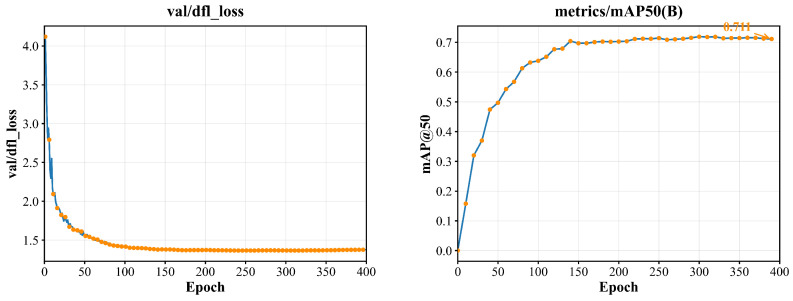
DFL loss convergence curve and mAP@50 evolution curve.

YOLOv12-VSD is trained systematically on the source domain. The resulting curves are shown in Figure 7.

As shown in Figure 7, the DFL loss decreases monotonically and converges to approximately 1.36 near epoch 245. mAP@50 reaches 0.711 by epoch 390 and stabilizes in the 0.71–0.72 range. The loss and accuracy curves converge smoothly in parallel, with no pronounced late-epoch oscillation. This pattern is consistent with the IoU-aware classification loss maintaining stable localization-aware confidence learning throughout training. Per-class evaluation results are shown in Table 4.

Per-class performance falls into three groups. Spidery Glass (mAP@50: 0.924), Crack (0.818), Inclusion (0.779), and Defaced Paint (0.719) are the top-performing categories. Their distinct edge contrast and moderate spatial scale are well matched to the multi-scale feature representations provided by P4-RepC3 and P5-SPPF. Paint-Peel (0.667) and Cutouts (0.636) sit in the middle tier, with adequate recall but limited high-IoU precision, a difficulty common to targets without sharply defined contours. Hairline Glass (0.630) and Clear Glass (0.550) remain the hardest classes because specular reflections and weak edge gradients reduce feature discriminability under standard RGB imaging conditions. The structural improvements proposed here address coarse-to-mid-scale defects effectively. Fine-grained specular categories remain the main area for further improvement.

**Figure 8:**
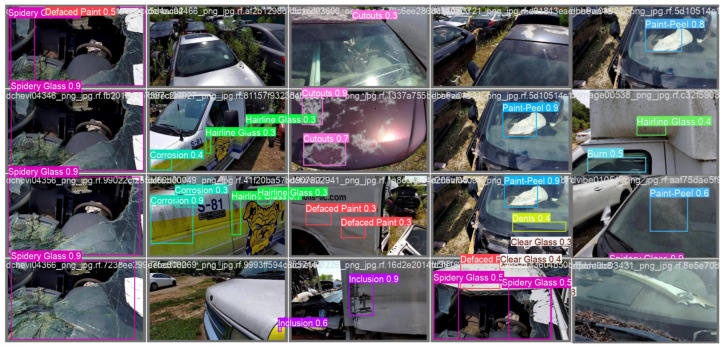
Detection results of the YOLOv12-VSD model. The rightmost column shows representative failure cases.

Detection results of the proposed model are shown in Figure 8.

The detection results in Figure 8 are consistent with the quantitative findings. Large-area defects are enclosed with high-confidence boxes, while hairline cracks and similar fine-scale targets receive lower but above-threshold scores that reflect genuine boundary uncertainty. The confidence values are well stratified across defect sizes, which is useful for production-line deployment where a single threshold must balance detection rate against false-alarm rate. The representative failure cases indicate that the remaining errors are mainly associated with reflective interference, bright non-defect structures, and partial occlusion. In these scenarios, subtle Hairline Glass patterns may be confused with Paint-Peel-like regions or suppressed by stronger local distractors. Thin linear non-glass traces may also occasionally trigger false positives.

### 4.4. Ablation Experiments

To verify the contribution of each proposed module, ablation experiments were conducted across all eight combinations of IoU-aware classification, P5-SPPF, and P4-RepC3. The complete results are summarized in Table 5.

Adding IoU-aware classification alone raises precision from 0.677 to 0.704 (+2.7 pp) at no additional inference cost (GFLOPs remain at 18.3). The gain is concentrated in boundary-sensitive categories, which is consistent with the idea that tying classification confidence to localization quality can suppress some high-confidence false positives. When structural modules are applied individually without IoU-aware classification, P5-SPPF alone reaches mAP@50 = 0.704 and P4-RepC3 alone reaches 0.696, both above the baseline (0.688). In this setting, P5-SPPF shows the larger standalone gain. Combining structural modules with IoU-aware classification is associated here with concurrent gains in precision and recall. IoU-aware classification combined with P4-RepC3 achieves mAP@50 = 0.698, while adding P5-SPPF to P4-RepC3 (without IoU-aware classification) reaches 0.710 with higher recall (*R* = 0.707). The full three-component model yields the most balanced overall result (*P* = 0.684, *R* = 0.697, mAP@50 = 0.715) and ranks highest among the evaluated configurations. The IoU-aware+P5-SPPF configuration achieves the highest mAP@50:95 (0.380), slightly above the full model (0.379). This 0.001 gap may indicate a mild precision–localization trade-off after adding P4-RepC3: recall rises from 0.689 to 0.697 and mAP@50 increases from 0.712 to 0.715, while the stricter mAP@50:95 decreases by 0.001. In the current experiments, this trade-off appears minor relative to the corresponding mAP@50 gain and the FPS improvement from 67.2 to 104.3. Despite the cumulative module additions, total GFLOPs drop from 18.3 to 17.1 in the full model. P4-RepC3 folds its multi-branch training convolutions into a single 3 × 3 kernel at inference, which helps offset the modest overhead introduced by P5-SPPF. This efficiency is also reflected in measured inference speed. YOLOv12-VSD reaches 104.3 FPS—the highest among all eight configurations—compared with 69.2 FPS for the unmodified baseline. Adding P5-SPPF alone reduces throughput slightly to 67.2 FPS, whereas the P4-RepC3+P5-SPPF combinations recover speed to 104.3 FPS. This pattern is consistent with the lower inference overhead of reparameterized convolution in this setting.

### 4.5. Comparison Experiments

To evaluate the overall competitiveness of YOLOv12-VSD, six representative improved YOLO models were used as comparison baselines. These models were reimplemented according to the corresponding original papers and retrained locally in a unified experimental pipeline. All models were trained and evaluated with identical settings to ensure a fair comparison. The results are recorded in Table 6.

For each model in Table 6, the second row reports the standard deviation (std) across N = 5 independent training runs with fixed seeds (42, 123, 456, 789, 1024). Bold values in the std rows mark the most stable result within each metric column.

As shown in Table 6, YOLOv12-VSD records the highest mAP@50 (0.715) and mAP@50:95 (0.379) among the compared methods. It also delivers the highest inference throughput at 104.3 FPS on a single NVIDIA RTX 4090. With 6.1 M parameters, the proposed model remains in the lightweight range of the compared methods, suggesting a favorable balance between accuracy and real-time latency in this setting.

Among the three models whose mAP@50 exceeds 0.710, YOLOv12-VSD shows clear advantages in both throughput and training reproducibility. LDBF-YOLO is the closest accuracy competitor (mAP@50 = 0.712, trailing by 0.003). However, its cross-run standard deviations for precision and recall (0.034 and 0.039) are approximately twice those of YOLOv12-VSD (0.017 and 0.008), which suggests greater sensitivity to random initialization across runs. Although LDBF-YOLO uses fewer parameters (4.7 M vs. 6.1 M) and a slightly lower computational budget (15.6 vs. 17.1 GFLOPs), it operates at 92.1 FPS, 13.2 percent below the proposed model. DART-YOLO reaches mAP@50 = 0.711 (trailing by 0.004) with 5.1 M parameters, yet its inference speed is only 55.8 FPS, approximately half that of YOLOv12-VSD. Its mAP@50 standard deviation (0.008) is also twice that of YOLOv12-VSD (0.004), which likewise suggests lower run-to-run stability in the current experiments.

The remaining four baselines show larger accuracy deficits. SCBF-YOLO (mAP@50 = 0.704) employs BiFPN with channel and spatial attention, uses 5.3 M parameters, and reaches 67.3 FPS in the current setting. TRS-YOLO (mAP@50 = 0.700) uses 7.5 M parameters and carries the highest computational load among all compared models (19.4 GFLOPs). It also exhibits a precision–recall imbalance (*P* = 0.716, *R* = 0.670), consistent with a higher-confidence but lower-recall operating profile. YOLOv12-VSD exceeds it by 0.015 in mAP@50, with a more balanced P/R profile and nearly twice the throughput (104.3 vs. 60.6 FPS). DCTL-YOLO (mAP@50 = 0.693) and FAST-YOLO (mAP@50 = 0.684) are both compact designs at 4.7 M parameters, yet their measured throughput remains 61.1 and 79.6 FPS, respectively. These comparisons indicate that parameter count alone does not determine deployment efficiency in the present setting. The proposed combination of module design and a reparameterized inference path is also important. Across all six baselines, YOLOv12-VSD records the lowest cross-run standard deviation for mAP@50 (0.004), indicating that its accuracy advantage is reproducible rather than appearing only with a single seed. The compact footprint (6.1 M parameters, 17.1 GFLOPs) therefore remains compatible with deployment on resource-constrained embedded vision hardware.

### 4.6. Transfer Framework Validation

**Figure 9:**
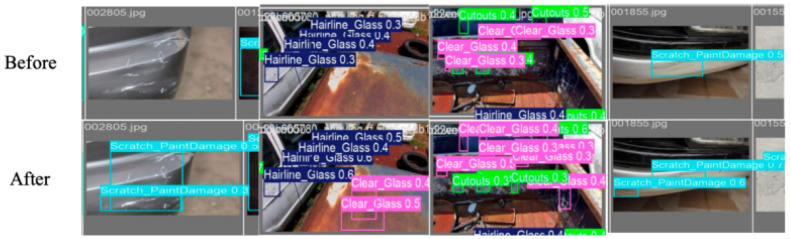
Comparison of detection results before and after fine-tuning.

Figure 9 provides a visual illustration of the quantitative trends above. Without target-domain adaptation, the model mislabels several instance types and produces fragmented, low-confidence detections, most visibly for fine-grained categories such as Hairline Glass and paint scratches. After Stage III fine-tuning, category labels are more often correctly assigned and predicted boxes appear tighter, consistent with the mAP@50:95/mAP@50 localization ratio increase from 0.476 to 0.525. Confidence scores also rise across many defect classes. Qualitative gains are most visible in spectrally subtle (Hairline Glass, Clear Glass) and geometrically irregular (Cutouts, Paint-Peel) categories, which is consistent with the recall-driven improvement in Table 7.

From the observed category-confusion patterns in the qualitative results, a common source of false positives before fine-tuning is cross-category misclassification between Hairline Glass and Clear Glass (overlapping spectral response for standard RGB imaging) and between Cutouts and Inclusion (similar concave contours and local texture gradients). Stage III fine-tuning appears to reduce these cross-category errors for both pairs. At the same time, the representative failure cases in Figure 8 indicate that the main remaining limitations are concentrated in scenes with reflective backgrounds, bright foreign objects, or partial occlusion, where the local appearance of thin defects can be distorted or visually suppressed. Under these conditions, Hairline Glass may still shift toward visually broader categories. Weak crack evidence may also remain difficult to preserve consistently.

#### 4.6.1. Transfer Learning Ablation Experiments

Three training configurations were compared. The results are shown in Table 8.

Switching from random to VSD.pt initialization improves mAP@50 by 2.0 pp (0.529 to 0.549) and reduces training time by 28.6% (1.301 h to 0.929 h). This pattern is consistent with the transferability of general defect feature representations learned in Stage I. The largest jump is observed after Stage III low-learning-rate fine-tuning: mAP@50 advances a further 10.7 pp to 0.656, and training time falls to 0.318 h. This may be related to Stage III starting from weights already close to the target optimum and therefore requiring only a brief refinement pass. Applying high learning rates directly to domain-shifted data may disrupt the general features acquired in Stage I. In the current experiments, the staged progression is associated with a more stable convergence trajectory.

#### 4.6.2. Transfer Learning Comparison Experiments

Six improved YOLO variants initialized from matching general pretrained checkpoints are compared with YOLOv12-VSD initialized from VSD.pt. The results are shown in Table 9.

Among all transfer learning baselines, YOLOv12-VSD records the highest mAP@50 (0.656), recall (0.596), and precision (0.668), leading the second-ranked SCBF-YOLO by 5.1 pp in mAP@50. The training-time gap is also large: YOLOv12-VSD completes fine-tuning in 0.318 h, compared to 3.241 h for TRS-YOLO, 3.473 h for LDBF-YOLO, and 7.135 h for DART-YOLO. This corresponds to a speedup of more than five times relative to those three models. Two factors may contribute to this gap. First, the compact architecture (6.1 M parameters, 17.1 GFLOPs) reduces per-epoch computation. Second, Stage III starts from weights already adapted by Stage II, so only a short refinement pass is required before convergence. The combined accuracy and efficiency profile is therefore consistent with the practical feasibility of deploying YOLOv12-VSD across different production scenarios with limited additional annotation effort.

## 5. Conclusions

This paper proposes YOLOv12-VSD and a coordinated three-stage transfer learning framework to address boundary localization inconsistency, insufficient multi-scale feature response, and cross-scenario generalization degradation in vehicle surface defect detection. On the source domain, YOLOv12-VSD improves mAP@50 from 0.688 to 0.715 over the baseline and records the highest mAP@50 among six comparison models while maintaining a compact 6.1 M-parameter, 17.1 GFLOP design. After three-stage transfer to the target domain, mean mAP@50 increases from 0.531 to 0.652, while training duration decreases from 0.794 h to 0.289 h. These results suggest that the proposed design is promising for balancing accuracy, efficiency, and transferability in this setting. Limitations include reliance on a single publicly available target-domain dataset and the absence of layer-freezing or knowledge distillation in the transfer pipeline. Future work will explore self-supervised pretraining, active learning for annotation reduction, and cross-dataset validation on public automotive defect benchmarks.

## Figures and Tables

**Table 1:** Summary of the proposed modifications relative to baseline YOLOv12.

Module	Position	Main Purpose	Cost Note
IoU-aware classification loss	Detection loss/classification supervision	Improve localization-aware confidence learning	Training only; no inference overhead
P4-RepC3	Neck, P4 feature layer	Strengthen mid-scale feature extraction	Reparameterized to a single convolution at inference
P5-SPPF	Neck, P5 feature layer	Improve high-level contextual aggregation	Slight additional inference computation
Three-stage transfer learning framework	Training pipeline	Improve cross-domain adaptation efficiency	No effect on the inference graph

**Table 2:** Simulation environment.

Item	Configuration
Operating System	Linux
CPU	Intel Xeon Gold 6430 (16 cores)
GPU	NVIDIA RTX 4090
Video Memory	24 GB
Python	3.12
PyTorch	2.5.1 + CUDA 12.4

**Table 3:** Training parameter configuration.

Parameter	Value	Description
Epochs	400	Total training epochs
Batch	16	Samples per minibatch
Imgsz	640	Input image resolution (pixels)
lr0	0.008	Initial learning rate
Lrf	0.01	Final learning-rate factor
Momentum	0.937	SGD momentum
Weight decay	0.0005	Weight decay coefficient
Patience	100	Early stopping patience
Seed	0	Random seed for reproducibility
Amp	True	Automatic mixed precision
Close_mosaic	10	Disable mosaic in the last 10 epochs
Box loss	7.5	Bounding-box loss gain
Cls loss	0.5	Classification loss gain
DFL loss	1.5	Distribution focal loss gain

**Table 4:** Detection metrics for each defect class.

Class	Precision	Recall	mAP@50	mAP@50:95
Clear Glass	0.56	0.527	0.55	0.237
Crack	0.821	0.789	0.818	0.414
Cutouts	0.608	0.644	0.636	0.285
Defaced Paint	0.685	0.73	0.719	0.425
Hairline Glass	0.656	0.544	0.63	0.332
Inclusion	0.700	0.731	0.779	0.385
Paint-Peel	0.621	0.714	0.667	0.303
Spidery Glass	0.820	0.898	0.924	0.649
Total	0.684	0.697	0.715	0.379

**Table 5:** Ablation experiment results. *P*: precision; *R*: recall. ✓: module enabled; –: module absent. Bold values indicate the best result achieved by YOLOv12-VSD across module configurations.

Method	IoU	P5	P4	*P*	*R*	mAP@50	mAP@50:95	GFLOPs	FPS
YOLOv12	–	–	–	0.677	0.658	0.688	0.369	18.3	69.2
	✓	–	–	0.704	0.649	0.694	0.364	18.3	69.2
	–	✓	–	0.668	0.698	0.704	0.373	18.9	67.2
	–	–	✓	0.669	0.666	0.696	0.369	16.5	83.4
	✓	–	✓	0.695	0.657	0.698	0.370	16.5	83.4
	–	✓	✓	0.658	0.707	0.710	0.377	17.1	104.3
	✓	✓	–	0.693	0.689	0.712	0.380	18.9	67.2
YOLOv12-VSD	✓	✓	✓	0.684	0.697	**0.715**	**0.379**	17.1	**104.3**

**Table 6:** Comparison experiment results. Italicized rows beneath each model report the standard deviation across N = 5 independent training runs with fixed seeds; bold values mark the best result in each metric column.

Method	*P*	*R*	mAP@50	mAP@50:95	Params (M)	GFLOPs	FPS
DART-YOLO	0.664	0.689	0.711	0.372	5.1	16.0	55.8
±*std*	±0.016	±0.014	±0.008	±0.007	—	—	—
LDBF-YOLO	0.673	0.702	0.712	0.376	4.7	15.6	92.1
±*std*	±0.034	±0.039	±0.012	±0.011	—	—	—
DCTL-YOLO	0.679	0.663	0.693	0.339	4.7	15.2	61.1
±*std*	±0.029	±0.013	±0.014	±0.012	—	—	—
FAST-YOLO	0.646	0.667	0.684	0.371	4.7	15.5	79.6
±*std*	±0.018	±0.012	±0.015	±0.013	—	—	—
SCBF-YOLO	0.656	0.687	0.704	0.377	5.3	16.0	67.3
±*std*	±0.018	±0.010	±0.013	±**0.005**	—	—	—
TRS-YOLO	0.716	0.670	0.700	0.374	7.5	19.4	60.6
±*std*	±0.021	±0.013	±0.010	±0.009	—	—	—
YOLOv12-VSD	0.684	0.697	0.715	0.379	6.1	17.1	**104.3**
±*std*	±0.017	±**0.008**	±**0.004**	±0.006	—	—	—

**Table 7:** Comparison of results before and after applying the transfer learning framework.

Trial	Before					After				
	***P***	***R***	**mAP@50**	**mAP@50:95**	**Dur.(h)**	***P***	***R***	**mAP@50**	**mAP@50:95**	**Dur.(h)**
1	0.660	0.501	0.531	0.254	0.814	0.675	0.583	0.659	0.344	0.307
2	0.625	0.505	0.528	0.251	0.791	0.677	0.580	0.651	0.341	0.308
3	0.670	0.458	0.531	0.254	0.780	0.632	0.592	0.647	0.340	0.293
4	0.670	0.457	0.530	0.253	0.788	0.681	0.591	0.659	0.345	0.280
5	0.658	0.485	0.529	0.252	0.799	0.618	0.592	0.645	0.340	0.257
Mean	0.657	0.481	0.531	0.253	0.794	0.657	0.588	0.652	0.342	0.289

**Table 8:** Performance comparison using transfer learning.

	*P* (%)	*R* (%)	mAP@50 (%)	Duration (h)
YOLOv12-VSD	0.653	0.510	0.529	1.301
YOLOv12-VSD + VSD.pt	0.638	0.525	0.549	0.929
YOLOv12-VSD + VSD.pt + Fine-tuning	0.668	0.596	0.656	0.318

**Table 9:** Performance comparison of each model.

Model + Weights	*P* (%)	*R* (%)	mAP@50 (%)	Duration (h)
SCBF-YOLO + s.pt	0.643	0.53	0.605	1.614
TRS-YOLO + m.pt	0.652	0.535	0.601	3.241
LDBF-YOLO + m.pt	0.632	0.515	0.594	3.473
DART-YOLO + l.pt	0.625	0.508	0.590	7.135
DCTL-YOLO + s.pt	0.615	0.490	0.578	1.486
FAST-YOLO + n.pt	0.598	0.470	0.565	0.615
YOLOv12-VSD + VSD.pt	0.668	0.596	0.656	0.318

## Data Availability

The source-domain dataset (NCAT12-DET) used in this study is publicly available at https://github.com/Brym-Gyimah/NCAT12-DET (accessed on 14 April 2026) [33]. The target-domain dataset was reorganized from the publicly available CarDD dataset [34] (https://cardd-ustc.github.io (accessed on 14 April 2026)); the reorganized subset is available on request from the corresponding author.

## Abbreviations

The following abbreviations are used in this manuscript:

YOLO	You Only Look Once
VSD	Vehicle Surface Defect
IoU	Intersection over Union
CIoU	Complete Intersection over Union
P4-RepC3	Reparameterized Convolution at P4 Layer
P5-SPPF	Spatial Pyramid Pooling–Fast at P5 Layer
SPPF	Spatial Pyramid Pooling Fast
mAP	Mean Average Precision
GFLOPs	Giga Floating-Point Operations
